# Timing of Restenosis and Reocclusion Following Endovascular Therapy for Femoropopliteal Lesions

**DOI:** 10.7759/cureus.107342

**Published:** 2026-04-19

**Authors:** Kenji Suzuki, Daisuke Ueshima, Kazuki Tobita, Naoki Hayakawa, Shinsuke Mori, Yo Iwata, Kazunori Horie, Tatsuya Nakama

**Affiliations:** 1 Department of Cardiology, Tokyo Saiseikai Central Hospital, Tokyo, JPN; 2 Department of Cardiology, Kameda Medical Center, Kamogawa, JPN; 3 Department of Cardiology, Shonan Kamakura General Hospital, Kamakura, JPN; 4 Department of Cardiovascular Medicine, Asahi General Hospital, Asahi, JPN; 5 Department of Cardiology, Saiseikai Yokohamashi Tobu Hospital, Yokohama, JPN; 6 Department of Cardiology, Funabashi Municipal Medical Center, Funabashi, JPN; 7 Department of Cardiology, Sendai Kousei Hospital, Sendai, JPN; 8 Department of Cardiology, Tokyo Bay Urayasu Ichikawa Medical Center, Urayasu, JPN

**Keywords:** evt: endovascular therapy, femoropopliteal artery, peripheral arterial diseases, reocclusion, restenosis

## Abstract

Purpose: Drug-eluting device and covered stent have become the mainstream of endovascular therapy for femoropopliteal lesions. Although there have been many reports on the primary patency of each device, there are few detailed data on restenosis and reocclusion.

Materials and methods: This was a physician-initiated, multicenter, retrospective study. From seven institutes, 3,635 femoropopliteal cases were enrolled in this study. Among them, we studied 2,786 cases treated with drug-eluting stent (DES), drug-coated balloon (DCB), covered stent (CS), and bare nitinol stent (BNS).

Results: The lesion background for each device was different, with mean lesion lengths of 140 (80-220) mm for BNS, 250 (180-300) mm for CS, 200 (100-260) mm for DES, and 123 (60-216) mm for DCB (p<0.001). Primary patency at two years was 62% for BNS, 75% for CS, 74% for DES, and 65% for DCB. The timing of restenosis and reocclusion was also different for each device. Peak restenosis timing was six to nine months for BNS and DCB and 12-15 months for CS and DES. The timing of reocclusion was six to nine months for BNS and DES, three to six months for CS, and zero to three months for DCB.

Conclusions: Not only was the primary patency for each device different, but the timing of restenosis and reocclusion for each device was also different.

## Introduction

Recently, drug technology and covered stent (CS) have been developed for the endovascular therapy (EVT) of peripheral arterial disease (PAD) affecting the femoropopliteal arteries. As a result, the European Society of Cardiology Guidelines recommend EVT for femoropopliteal lesions < 25 cm in size [1]. Since 2006, bare nitinol stent (BNS) has improved primary patency compared to balloon dilatation, but restenosis rates remained high [2,3]. Then, in 2008, a favorable result for drug-coated balloon (DCB) was reported [4]; thereafter, the effectiveness of DCB has been reported in many cases [5,6]. Similar to DCB, a drug-eluting stent (DES) reported good results [7], and over a five-year period, the effectiveness of primary patency compared to BNS was added [8]. It has also been reported that CS showed favorable outcomes for long lesions, and these results were cited in the guidelines [9].

Primary patency at one year for each device is often reported to be around 80% [7,10], but CS and DES are often used for longer and chronic total occlusion (CTO) lesion and have been reported to have higher primary patency [11,12]. In general, CS and DES seem to have better results; however, DCB is often the first choice for treatment of femoropopliteal lesions, as represented by “leave nothing behind strategy.” One reason may be that reocclusion often worsens the condition. Second, restenosis is often easy to treat and has a good prognosis, while reocclusion is not only difficult to open but also has a poor prognosis [13-17].

There are several reports on how to prevent reocclusion. The first is the use of interventional tips, and the second is the use of antithrombotic agents [18-20]. However, because of the problem of bleeding complications, the duration of antithrombotic agents should be shortened. This study aimed to compare the timing patterns of restenosis and reocclusion following endovascular therapy for femoropopliteal lesions, focusing on differences among DES, DCB, CS, and BNS. This article was previously posted on the Research Square preprint server on November 09, 2025.

## Materials and methods

Study design

The reStenosis and reoCclusiOn timing of endovascular theRapy for femoroPopliteal lesIONs (SCORPION) study is a multicenter, retrospective analysis of a prospectively maintained database. A total of 3,635 patients with symptomatic peripheral artery disease (Rutherford categories 2 to 6) underwent femoropopliteal EVT between January 2017 and February 2021 at seven cardiovascular centers. A total of 2,786 cases with DES, DCB, BNS, and CS as finalizing devices were studied.

Treatment procedure

Dual antiplatelet therapy was administered before the procedure. Dual antiplatelet therapy was continued a minimum of two months after deployment of DES, a minimum of six months after deployment of CS, and a minimum of one month after use of DCB and BNS. The intervention procedure and device selection were performed at the operator's discretion. The indications for intervention included Rutherford grades 2-6 cases with >50% diameter stenosis determined by angiography. After unfractionated heparin was injected, a 5F to 7F sheath was introduced. A 0.014- to 0.035-inch guidewire was advanced into the lesion, and balloon dilation was performed. Stent deployment was recommended if there was severe dissection, described as National Heart, Lung, and Blood Institute (NHBLI) D-to-F, or ≥50% residual stenosis. When a mobile thrombus was detected, a stent was placed or thrombus aspiration was performed. The post-treatment follow-up and the patency of the target vessel were evaluated using duplex sonography, computed tomography (CT), or angiography, depending on the recommendations of the Society of Vascular Surgery, every three months during the first year, every six months, or annually thereafter.

Outcome measures and definitions

The primary endpoint is the rate of restenosis or reocclusion every three months up to two years. The secondary outcome measure was primary patency, death, and major adverse limb event. Restenosis was defined as a ratio of peak systolic velocity ≥2.5 on duplex sonography or vessels with ≥50% diameter stenosis, and reocclusion was defined as no color Doppler on duplex or no contrast inflow on CT or contrast in the stent area. BNS included both regular self-expand nitinol stents and SUPERA stent (Abbott Laboratories, Abbott Park, IL, USA). DES was defined as the ELUVIA stent (Boston Scientific, Marlborough, MA, USA) and the ZILVER PTX stent (Cook Corporation, Bloomington, IN, USA). DCBs could enroll all DCBs available in Japan.

Statistical analysis

Data on baseline characteristics are presented as the mean ± standard deviation (SD) for continuous variables and the frequency (percentage) for categorical variables, if not otherwise mentioned. p <0.05 was considered significant, and 95% confidence intervals (CIs) were reported as appropriate. In this study, we represented the incidence rates of occlusion and restenosis using histograms, while employing probability density functions to visualize the probabilistic distribution of the timing of each event's occurrence. The probability density function does not directly indicate the probability of a specific value itself, but rather represents the probability that data falls within a certain range. For example, the probability that a measurement falls within a specific range can be determined by integrating the value of the probability density function over that range. Additionally, the probability density function is defined such that its integral over all possible values equals 1.

To flexibly estimate the shape of the probability density function without assuming a prior distribution of the data, we employed Kernel Density Estimation. We executed the Kernel Density Estimation using the "geom_density()" function from the R "ggplot2" package. The "geom_density()" function internally utilizes the "density()" function, employing the Gaussian kernel as the kernel function and automatically calculating the bandwidth based on Silverman’s Rule of Thumb. Kernel Density Estimation is utilized in conjunction with histograms to visualize the data distribution more clearly. In particular, it enables a smooth visualization of the distribution that is less affected by the bins of the histogram, allowing for an intuitive understanding of the characteristics of the data.

## Results

From the total of 3.635 patients, 699 were in the BNS group, 211 in the CS group, 550 in the DES group, and 1,326 in the DCB group. Cases with multiple devices, such as a mixture of DES and CS, were excluded. The baseline patient characteristics of the patients are shown in Table 1. Significant differences were found in several parameters, such as more women in the CS group, more Cilostazol administration, and less dyslipidemia. The baseline lesion and procedure characteristics of the patients are shown in Table 2. Lesion length and chronic total occlusion (CTO) rates were 140 mm and 49.9% in the BNS group, 250 mm and 69.2% in the CS group, 200 mm and 56.9% in the DES group, and 123 mm and 26.0% in the DCB group. Some parameters, such as cases involving the popliteal artery and vessel diameter, were also indicated to show significant differences. However, we did not perform propensity match analysis as well as patient background to show the characteristics in real-world data.

**Table 1:** Baseline patient characteristics Categorical variables are expressed as numbers and percentages. Continuous variables are indicated as mean ± standard deviation or median (interquartile range). BNS = bare nitinol stent, CS = covered stent, DCB = drug-coated balloon, DES = drug-eluting stent, eGFR = estimated glomerular filtration rate, CAPD = continuous ambulatory peritoneal dialysis.

	BNS group	CS group	DES group	DCB group	P value
n	699	211	550	1,326	
Gender	533 (76.3%)	130 (61.6%)	402 (73.1%)	923 (69.6%)	<0.001
Age (years)	74.9 ± 9.2	75.5 ± 9.1	74.7 ±9 .0	73.9 ± 9.2	0.015
Body mass index (kg/m^2^)	22.8 ± 4.0	22.1 ± 3.5	22.4 ± 3.9	22.7 ± 4.0	0.050
Ambulatory	580 (83.0%)	176 (83.7%)	467 (84.9%)	1,108 (83.6%)	0.829
Hypertension	570 (81.5%)	179 (84.8%)	458 (83.4%)	1,094 (82.5%)	0.672
Diabetes mellitus	453 (64.8%)	119 (56.4%)	356 (64.7%)	876 (66.1%)	0.059
Hyperlipidemia	454 (64.9%)	104 (49.3%)	353 (64.2%)	867 (65.4%)	<0.001
Smoking (current and past)	505 (72.5%)	128 (64.0%)	406 (73.8%)	934 (70.5%)	0.001
Chronic kidney disease					0.004
eGFR<60	214 (30.6%)	89 (42.2%)	216 (39.3%)	473 (35.7%)	
Renal failure on dialysis	238 (34.0%)	57 (27.0%)	158 (28.7%)	467 (35.7%)	
CAPD	1 (0.1%)	1 (0.5%)	2 (0.4%)	9 (0.7%)	
Coronary artery disease	349 (49.9%)	116 (55.0%)	269 (48.9%)	665 (50.2%)	0.510
Cerebral vascular disease	147 (21.0%)	38 (18.0%)	123 (22.4%)	324 (24.4%)	0.108
Heart failure	165 (23.6%)	31 (14.7%)	120 (21.8%)	299 (22.5%)	0.050
Atrial fibrillation	117 (16.7%)	35 (16.6%)	88 (16.0%)	203 (15.3%)	0.851
Aspirin	554 (79.3%)	178 (84.4%)	430 (78.2%)	1,013 (76.4%)	0.054
Thienopyridine	586 (83.8%)	183 (86.7%)	481 (87.5%)	1,126 (84.9%)	0.292
Cilostazol	191 (27.3%)	107 (50.7%)	169 (30.7%)	348 (26.2%)	<0.001
Anti-coagulation	112 (16.0%)	42 (19.9%)	84 (15.3%)	219 (16.5%)	0.478
Double anti-platelet therapy	478 (68.3%)	161 (76.3%)	374 (68.0%)	868 (65.5%)	0.017
Anti-platelet plus anti-coagulation	104 (14.9%)	35 (16.6%)	78 (14.2%)	191 (14.4%)	0.839
Rutherford classification					0.001
Category 2	78 (11.2%)	51 (24.2%)	59 (10.7%)	160 (12.1%)	
Category 3	414 (46.0%)	101 (47.9%)	320 (58.2%)	768 (57.9%)	
Category 4	67 (9.6%)	24 (11.4%)	53 (9.6%)	117 (8.8%)	
Category 5	125 (17.9%)	31 (14.7%)	105 (19.1%)	250 (18.9%)	
Category 6	15 (2.1%)	4 (1.9%)	13 (2.4%)	31 (2.3%)	
Ankle brachial index	0.62 (0.50, 0.73)	0.60 (0.49, 0.72)	0.63 (0.50, 0.74)	0.67 (0.54, 0.80)	<0.001

**Table 2:** Baseline lesion characteristics of study population Categorical variables are expressed as number and percentage. Continuous variables are indicated as mean ± standard deviation or median (interquartile range). BNS = bare nitinol stent, CS = covered stent, DCB = drug-coated balloon, DES = drug-eluting stent, SFA = superficial femoral artery, CTO = chronic total occlusion, PACSS = Peripheral Arterial Calcium Scoring System, EVUS = extravascular ultrasound, IVUS = intravascular ultrasound.

	BNS group	CS group	DES group	DCB group	P value
n	699	211	550	1,326	
Lesion location					<0.001
SFA	539 (77.1%)	92 (43.6%)	382 (69.5%)	775 (58.4%)	
Popliteal	65 (9.3%)	12 (5.7%)	105 (19.1%)	240 (18.1%)	
SFA to popliteal	95 (13.6%)	107 (50.7%)	147 (26.7%)	311 (23.5%)	
De novo	582 (83.3%)	151 (71.6%)	468 (85.1%)	986 (74.4%)	<0.001
Reference diameter (IQR)	5.5 (5.0-6.0)	5.8 (5.5-6.0)	5.5 (5.0-6.0)	5.2 (4.7-5.8)	<0.001
Lesion length (IQR)	140 (80-220)	250 (180-300)	200 (100-260)	123 (60-216)	<0.001
CTO	349 (49.9%)	146 (69.2%)	398 (56.9%)	345 (26.0%)	<0.001
CTO length (IQR)	150 (60-220)	200 (90-250)	190 (90-250)	100 (42-210)	<0.001
PACSS grade					<0.001
0	230 (32.9%)	76 (36.0%)	205 (37.3%)	551 (41.6%)	
1	68 (9.7%)	33 (15.6%)	66 (12.0%)	183 (13.8%)	
2	30 (4.3%)	12 (5.7%)	38 (6.9%)	81 (6.1%)	
3	103 (14.7%)	21 (10.0%)	71 (12.9%)	179 (13.5%)	
4	268 (38.3%)	69 (32.7%)	170 (30.9%)	332 (25.0%)	
Wire crossing					<0.001
Fluoro	565 (80.8%)	182 (86.3%)	441 (80.2%)	1195 (90.1%)	
EVUS	36 (5.2%)	19 (9.0%)	32 (5.8%)	38 (2.9%)	
IVUS	98 (14.0%)	10 (4.7%)	77 (14.0%)	93 (7.0%)	
Treatment device					<0.001
SUPERA	211 (30.2%)	0 (0.0%)	0 (0.0%)	0 (0.0%)	
BNS	488 (69.8%)	0 (0.0%)	0 (0.0%)	0 (0.0%)	
VIABAHN	0 (0.0%)	211 (100.0%)	0 (0.0%)	0 (0.0%)	
IN.PACT	0 (0.0%)	0 (0.0%)	0 (0.0%)	839 (63.3%)	
LUTONIX	0 (0.0%)	0 (0.0%)	0 (0.0%)	465 (35.1%)	
RANGER	0 (0.0%)	0 (0.0%)	0 (0.0%)	9 (0.7%)	
Other DCB	0 (0.0%)	0 (0.0%)	0 (0.0%)	13 (1.0%)	
ELUVIA	0 (0.0%)	0 (0.0%)	405 (73.6%)	0 (0.0%)	
ZILVER PTX	0 (0.0%)	0 (0.0%)	145 (26.4%)	0 (0.0%)	
Bail out stent	-	-	-	33 (2.5%)	
Complications					0.001
None	677 (96.9%)	198 (93.8%)	520 (94.5%)	1,307 (98.6%)	
Blood transfusion	2 (0.3%)	1 (0.5%)	1 (0.2%)	1 (0.1%)	
Distal embolization	6 (0.9%)	4 (1.9%)	7 (1.3%)	7 (0.5%)	
Perforation	6 (0.9%)	5 (2.4%)	11 (2.0%)	6 (0.5%)	
Puncture site trouble	8 (1.1%)	3 (1.4%)	11 (2.0%)	5 (0.4%)	

**Figure 1:**
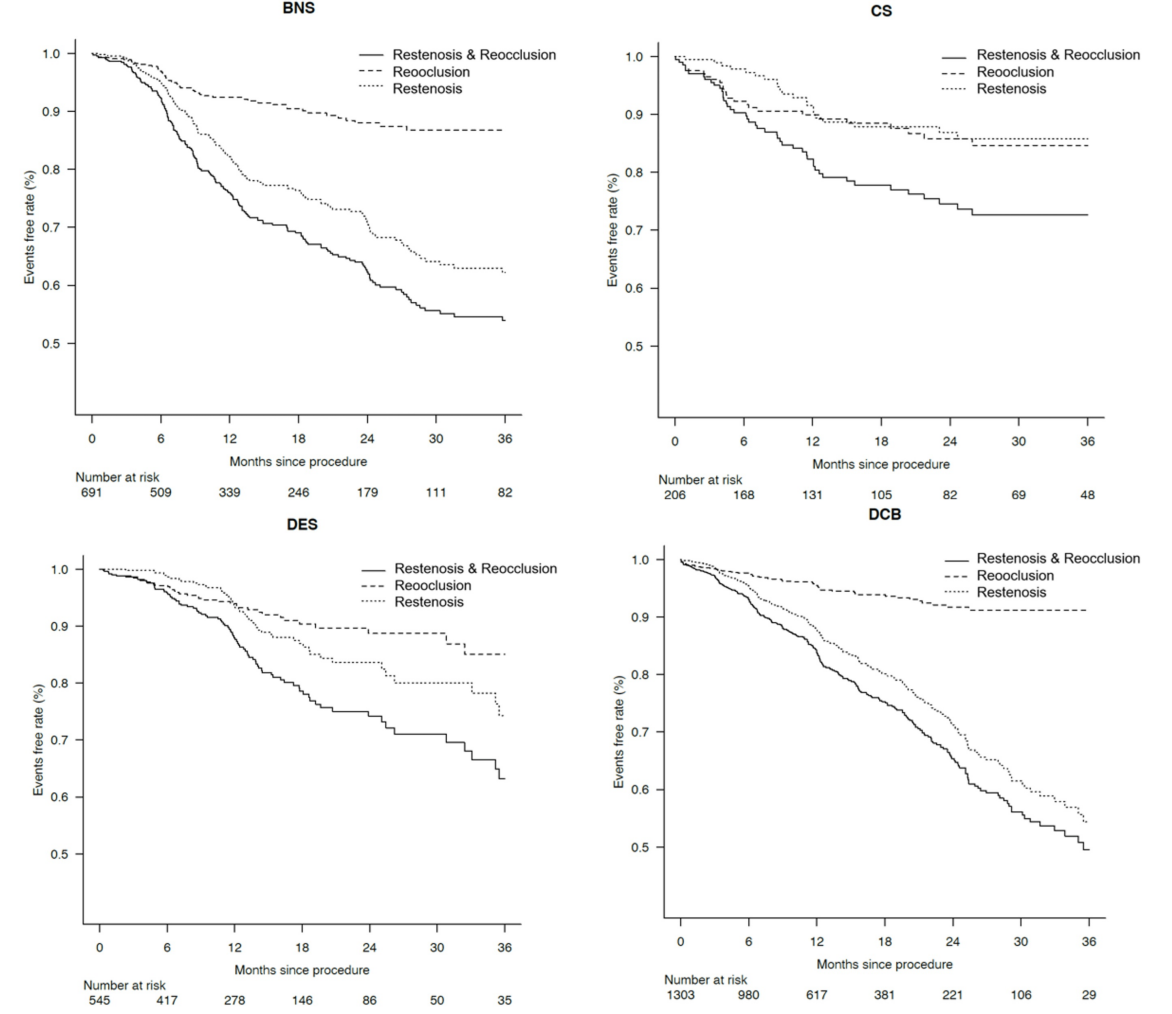
Kaplan-Meier curves showing reocclusion and restenosis for each device BNS = bare nitinol stent, CS = covered stent, DCB = drug-coated balloon, DES = drug-eluting stent.

**Figure 2:**
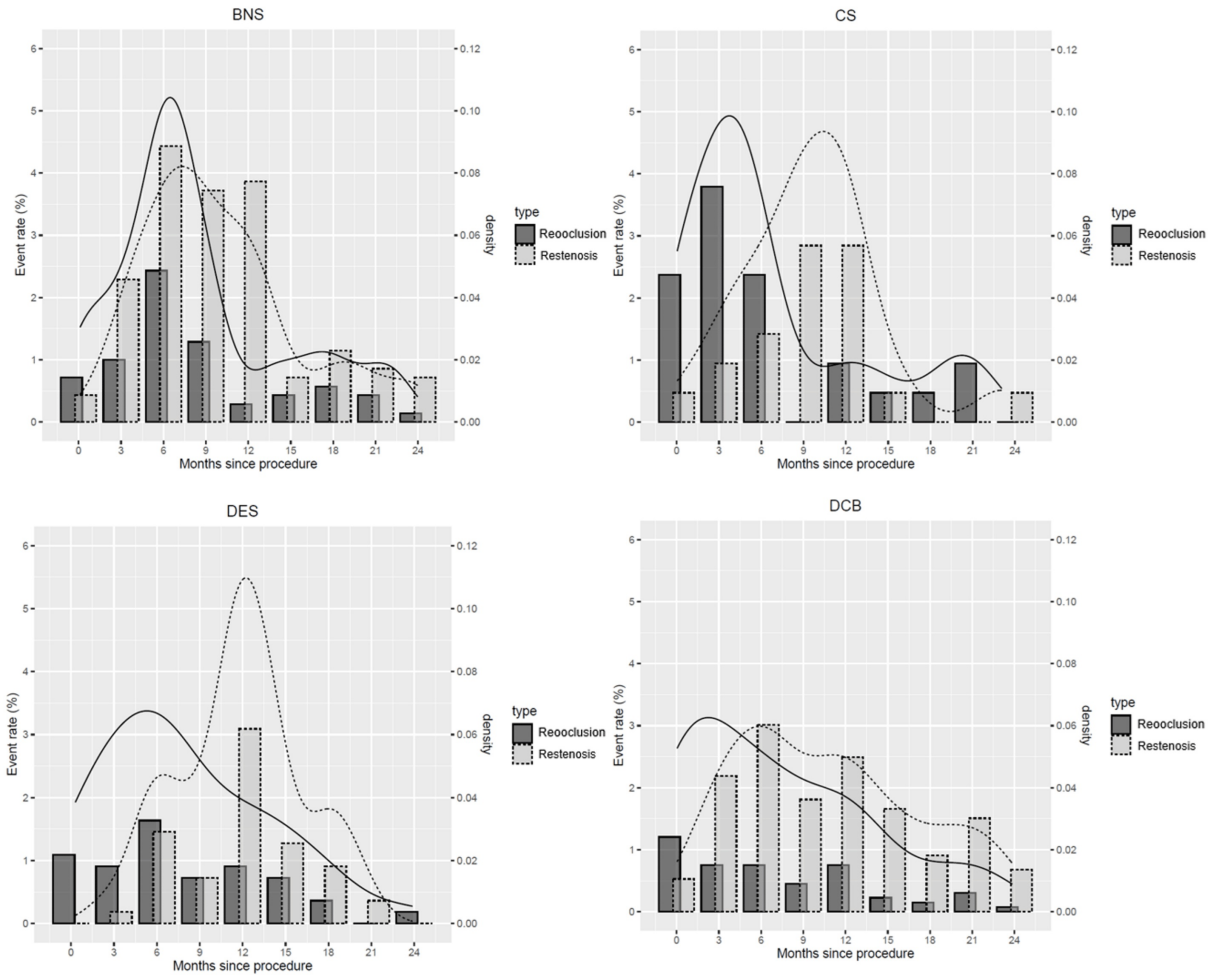
Number of reocclusion and restenosis every three months BNS = bare nitinol stent, CS = covered stent, DCB = drug-coated balloon, DES = drug-eluting stent.

Kaplan-Meier curves of primary patency divided into reocclusion and restenosis were shown in Figure 1. Because of the different lesion backgrounds, simple comparisons cannot be made, but the characteristics of restenosis for each device can be found. To clarify these characteristics, separate graphs were made for reocclusion and restenosis every three months (Figure 2). Peak restenosis timing was six to nine months for BNS and DCB and 12-15 months for CS and DES. The timing of reocclusion was six to nine months for BNS and DES, three-six months for CS, and zero to three months for DCB. The DCB group has fewer reocclusion, but a relatively high rate of restenosis is present; the DES and CS groups show a relatively same pattern. Particularly within six months, there is more reocclusion than restenosis, with a clear peak in restenosis at around one year.

Clinical results were presented in Table 3. CS, DCB, and DES had better freedom from target lesion revascularization (TLR) rate than BNS, but only DES was significantly better at two years. There were no significant differences in major amputation and mortality between the four groups.

**Table 3:** Clinical outcomes BNS = bare nitinol stent, CS = covered stent, DCB = drug-coated balloon, DES = drug-eluting stent, TLR = target lesion revascularization, CI = confidence interval.

	BNS group	CS group	DES group	DCB group
	699	211	550	1,326
TLR				
1y event free rate (95%CI). %	83 (80-86)	85 (79-89)	91 (88-94)	89 (87-91)
2y event free rate (95%CI). %	72 (68-76)	78 (70-83)	79 (74-84)	75 (71-78)
Hazard ratio (95% CI) vs. BNS	Control	0.72 (0.50-1.02)	0.72 (0.54-0.96)	0.90 (0.72-1.11)
Major amputation				
1y event free rate (95% CI) %	98 (96-99)	100 (96-100)	98 (96-99)	98 (97-99)
2y event free rate (95% CI) %	97 (95-98)	99 (94-100)	97 (95-98)	97 (95-98)
Hazard ratio (95% CI) vs. BNS	Control	0.36 (0.08-1.56)	1.04 (0.50-2.17)	0.91 (0.49-1.68)
All cause death				
1y event free rate (95% CI) %	88 (85-90)	93 (88-96)	90 (87-92)	91 (89-92)
2y event free rate (95% CI) %	-79 (76-83)	83 (76-88)	81 (76-85)	82 (79-85)
Hazard ratio (95% CI) vs. BNS	Control	0.85 (0.61-1.20)	0.93 (0.71-1.22)	0.87 (0.70-1.09)

## Discussion

This study examined the performance of various devices in real-world data, categorized by restenosis, reocclusion, and detailed timing. Many studies have already been reported on the results of each device, but there are no reports of detailed data focusing on reocclusion, restenosis, or timing. In general, BNS experiences patency loss in six months to one year, followed by a slow decline, while DCB is less likely to plateau after one year [7,21]. It is important to note that the DES and CS groups show relatively similar patterns, with patency loss within six months being mainly due to reocclusion rather than restenosis.

The key is to avoid reocclusion rather than restenosis. Since reocclusion is associated with extremity inferior long-term results [16] before the drug device was available. Even with the availability of drug devices, results for reocclusion have not been satisfactory [22]. In Japan, where there are few debulking devices available, the high rate of complications, including distal embolization, is also a concern [13]. Moreover, reocclusion treatment is likely to increase stent length [23]. It is therefore crucial to avoid reocclusion, and it has been reported that avoiding scaffold placement in small vessels, plaque landings, and popliteal lesions is important [24]. Scaffolds are reported to cause more thrombosis than DCBs, and acute limb ischemia (ALI) is also reported to increase [17]. However, no significant difference in major amputation (MA) rates was found in this study. Another method to improve patency is pharmacotherapy. In terms of improving long-term result of EVT, there have been several reports with cilostazol [25,26]. However, these are mainly limited to scaffold data, and DCB data are scarce.

In addition, the efficacy of anticoagulation therapy for PAD has been reported, particularly reducing acute limb ischemia [18,19]. In the latest guidelines, anticoagulation is strongly recommended as class of recommendation, Class I, level of evidence, Level A [27]. Thus, anticoagulants have the potential to reduce reocclusion, but DES need a double antiplatelet therapy (DAPT) period of more than two months and CS more than six months. The problem is the long-term use of triple antithrombotic agents. Sub-analysis suggest that DAPT plus anticoagulation for more than 30 days is associated with increased bleeding complications [20]. lower extremity artery disease (LEAD) patients are often at high bleeding risk, and concerns remain about long-term antithrombotic drug administration [28]. Anticoagulation only during periods of high reocclusion with scaffold implantation might lead to improved outcomes. Hence, it is extremely important to know when reocclusions are most common for each device.

In this study, devices were divided into four categories. Although there are reports that SUPERA stent have different patency rates than regular BNS and that ZILVER PTX and ELUVIA have different reocclusion rates [21, 29]. However, too detailed a classification could undermine the essence of this study, so four classifications were used.

DCB was the most used in the study, with 1,326 cases. Despite having the shortest lesion length as well as the fewest CTOs, primary patency is not the best. The fact that DCB is the most used despite low patency in easy lesions may indicate that reocclusion avoidance is a strong factor in device selection. The key point about patency loss in DCB is that reocclusions were most common in the early stages of treatment. This is most likely not an effect of the drug failure, but a so-called balloon failure. Confirmation of minimal lesion area with intravascular ultrasound [30], correct evaluation of dissection, and bail out stenting may contribute to improved DCB outcomes.

This study has several limitations. First, this study had a multicenter, retrospective, and non-randomized design, causing potential bias. Second, propensity match was not performed; there are differences in factors that may be associated with the restenosis rate, such as lesion length and restenotic lesions, and propensity match may actually distort the research results. Third, the exact duration of drug administration remains unknown and the data are from a period when low-dose rivaroxaban was not available. Fourth, data from multiple devices, such as DCB and DES combination, was excluded. Lastly, clinical follow-ups were performed at each institute; however, no follow-up protocol was established during the study period. To provide a simple overview of the characteristics of reocclusion and restenosis in these four types of devices, we must note that there are many issues to consider, such as procedural details, differences between devices, and variations in lesion backgrounds.

## Conclusions

This study classified cases of reocclusion and restenosis and analyzed detailed data collected every three months. Not only was the primary patency for each device different, but the timing of restenosis and reocclusion for each device was also different. Although validation in a prospective trial is required, in order to improve device performance, it will be necessary to understand the timing of restenosis and reocclusion.
